# Impact of Chronobiological Variation in Takotsubo Syndrome: Prognosis and Outcome

**DOI:** 10.3389/fcvm.2021.676950

**Published:** 2021-08-26

**Authors:** Ibrahim El-Battrawy, Assem Aweimer, Siegfried Lang, Uzair Ansari, Thorsten Gietzen, Niklas Ullrich, Andreas Mügge, Xiaobo Zhou, Martin Borggrefe, Ibrahim Akin

**Affiliations:** ^1^University of Mannheim, Mannheim, Germany; ^2^Department of Cardiology and Angiology, Bergmannsheil University Hospitals, Ruhr University of Bochum, Bochum, Germany

**Keywords:** takotsubo, chronobiology, heart failure, outcome, complication

## Abstract

**Background:** A considerable amount of evidence has shown that acute cardiovascular diseases exhibit specific temporal patterns in their onset.

**Aim:** This study was performed to determine if takotsubo syndrome (TTS) shows chronobiological variations with short and long-term impacts on adverse events.

**Design:** Our institutional database constituted a collective of 114 consecutive TTS patients between 2003 and 2015.

**Methods:** Patients were divided into groups defined by the onset of TTS as per time of the day, day of the week, month and quarter of year.

**Results:** TTS events were most common afternoon and least common in the night, indicating a wave-like pattern (*p* = 0.001) of manifestation. The occurrence of TTS events was similar among days of the week and weeks of the month. TTS patients diagnosed in the month of November and subsequently in the fourth quarter showed a significantly longer QTc interval. These patients also revealed a significantly lower event-free-survival over a 1-year follow-up. In a multivariate Cox regression analysis, TTS events occurring in the fourth quarter of year (HR 6.8, 95%CI: 1.3–35.9; *p* = 0.02) proved to be an independent predictor of lower event-free-survival.

**Conclusions:** TTS seems to exhibit temporal preference in its onset, but nevertheless this possibly coincidental result needs to be analyzed in a large multicenter registry.

## Introduction

The exact pathophysiological mechanism for selective wall motion abnormality in the absence of significant coronary artery stenosis remains unknown in Takotsubo syndrome (TTS). TTS patients may suffer from complications e.g., acute heart failure, ventricular tachyarrhythmias, thromboembolic events and significant mitral valve regurgitation similar to acute coronary syndrome (ACS). Patients often complain about chest pain and/or dyspnea (1–5). TTS has been firstly described 1991. TTS related complications may cause a similar mortality rate as ACS.

Chronobiology is the biomedical science aimed at the study of biological rhythms. Defined by the cycle length, biological rhythms may be divided into three main types: ultradian (period <24 h e.g., hours), circadian (24 h period e.g., days), infradian (>24 h e.g., weeks, months). The natural predilection of the cardiovascular system is oriented to function in an oscillatory circadian order, both in conditions of health or disease. The occurrence of cardiovascular events is not evenly distributed in time but shows peculiar temporal patterns that vary with time of the day, day of the week, and month of the year (6, 7).

There have been reports suggesting a chronobiological difference and circadian variation to the occurrence of TTS (8, 9). However, a description detailing the short and long-term impact of time onset of TTS on adverse events or complications is constrained by the lack of scientific data.

The primary hypothesis of the present study is that circadian variant may have a different impact on the outcome of TTS patients.

## Methods

We retrospectively studied a collective of 114 consecutive patients diagnosed with TTS between January 2003 and September 2015 at our institution, the Medical Faculty of Mannheim. Patients were diagnosed as per the Mayo Clinic Criteria (10), which outlines the clinical features associated with TTS, and the results were reviewed by two independent experienced cardiologists to reaffirm this diagnosis. Patients with a concomitant coronary artery disease were not excluded, but we excluded only patients with a significant coronary plaque, which explains the reason for wall motion abnormality. Eighteen patients with uncertain TTS (due to absence of coronary angiogram and/or follow-up echocardiogram) were excluded from this study.

This study was conducted in compliance with the Declaration of Helsinki concerning investigations in human subjects and the study protocol was approved by the Ethics Committee of the Medical Faculty Mannheim, University of Heidelberg. The need for informed consent was not required by the ethics committee. All methods were performed in accordance with the relevant guidelines and regulations.

Screening for TTS was done prospectively at the TTS event. The assessment of complications associated with TTS was conducted at index-event and at the subsequent follow-up after 1 year. The clinical outcome of patients was assessed by chart review and/or telephone review. If medical records, treating physicians or relatives were unable to substantiate information identifying the circumstances of death, it was defined as death due to an unknown cause.

All patients included in this study were grouped in a standardized fashion as per the timing of their initial 12-lead ECG recordings on admission to the emergency department reflecting the event onset and diagnosis of TTS. This was used to determine if the occurrence of this syndrome was consistent with circadian dependence. The day was spitted into 6-h blocks beginning at 06:00 a.m. The week was divided into 7 days and the year into 12 months. The year was divided into 3-month blocks: spring including March to May, summer including June to August, autumn including September to November, and finally winter including December to February.

The end-point of our study was a composite of thromboembolic events, life-threatening arrhythmias, all-cause mortality, re-hospitalization due to heart failure, stroke, myocardial infarction, and recurrence of TTS as assessed by chart review and/or telephone review over a mean follow-up for 1 year. The selection of endpoint components was based on previous results which have described these complications to influence the prognosis of TTS patients (11). The follow-up was 1 year.

## Statistics

Our data are presented as means ± SD for continuous variables with a normal distribution, median (interquartile range) for continuous variables with a non-normal distribution, and as frequency (%) for categorical variables. Student's *t*-test and the Mann–Whitney U-test were used to compare continuous variables with normal and non-normal distributions. The chi-squared-test and Fisher's exact test were used to compare categorical variables. Additionally, with the use of the chi-squared “Goodness of Fit” test, observed vs. expected numbers were compared to evaluate whether TTS events are evenly distributed across the day, months, or quarter of year. The log-rank test was used to compare the survival curves between groups. Factors with *p* < 0.10 in univariate analysis were subjected to the Cox multivariate regression analysis to define independent risk factors for the adverse outcome. Data was analyzed in an exploratory manner to generate new hypotheses and therefore we did not adjust the *p*-values with the number of all performed analyses. The IBM SPSS 23.0 or GraphPad InStat were used for statistical calculations. In all analyses, *p* < 0.05 (two-tailed) was taken to indicate statistical significance, 0.05 > *p* ≤ 0.10 was taken to indicate significance by tendency.

## Results

### Clinical Features of TTS Patients

The mean age of patients presenting with TTS was 67 ± 11 years, with our data skewing toward a female preponderance (83%), Table 1. The most common clinical symptom was chest pain (50.8%), followed by dyspnea (37%). An ST-segment elevation on ECG was observed in 30% and inverted T-waves in 89.5%. A detailed patient history revealed emotional stress in 29% and physical stress in 56% of the patients. LV-function at the time of admission, as measured by transthoracic echocardiography and laevo-cardiography, was moderately reduced (EF 38.3%). The involvement of the right ventricle was documented in 22.8% of the patients. An apical type TTS was observed in 72% (*n* = 82) of patients, whereas the non-apical ballooning form could be diagnosed in 28% (*n* = 32). The most frequent complications associated with TTS were life-threatening arrhythmias (11.4%), pulmonary congestion with need for invasive respiratory support (20%), thromboembolic events (12.2%), cardiogenic shock (19.2%) and in-hospital death (7.9%).

**Table 1:** Baseline characteristics of patients presenting with TTS in the fourth quarter of year.

**Variables**	**All quarters (*n* = 114)**	**Quarter 1–3 (*n* = 81)**	**Quarter 4 (*n* = 33)**	*p*-value^*^
**Demographics**				
Age, mean ± SD	67 ± 11	67.3 ± 10.9	66.8 ± 12.1	0.86
Female, *n* (%)	95 (83)	65 (80.24)	30 (90.90)	0.16
**Symptoms**, ***n*****(%)**				
Dyspnoe	58 (50.8)	34 (41.97)	9 (27.27)	0.14
Chest pain	43 (37)	41 (50.61)	17 (51.51)	0.93
**Clinic parameter**				
Systolic BP, mmHg	131.8 ± 33	134.07 ± 35.10	124.38 ± 22.03	0.17
Diastolic BP, mmHg	76.7 ± 17.5	78.07 ± 18.42	72.22 ± 14.42	0.22
Heart rate, bpm	100.2 ± 27.2	102.19 ± 27.03	96.59 ± 27.53	0.34
**ECG Data**, ***n*****(%)**				
ST-segment elevation	34 (30)	21 (25.92)	13 (39.39)	0.15
Inversed T-Waves	102 (89.5)	73 (90.12)	29 (87.87)	0.21
PQ-interval	160.5 ± 20.1	160.39 ± 30.65	160.92 ± 25.13	0.93
QTc (ms), mean ± SD	474 ± 69	472.96 ± 48.26	494.59 ± 59.59	0.05
**Stress factor**, ***n*****(%)**				
Emotional sress	30 (26.3)	24 (29.62)	6 (18.18)	0.20
Physical stress	64 (56.1)	46 (56.79)	18 (54.54)	0.82
None	25 (21.9)	16 (19.75)	9 (27.27)	0.37
**Laboratory values, mean ± SD**				
Troponin I (U/L)	3.6 ± 5.2	4.17 ± 5.86	2.70 ± 3.79	0.21
Creatine phosphatkinase (U/L)	640 ± 2,609	802.99 ± 3,129.13	288.16 ± 406.62	0.35
CKMB	35.8 ± 59.3	42.48 ± 70.82	22.17 ± 14.48	0.24
C-Reactive protein (mg/l)	49 ± 78.4	55.5 ± 88.2	33.7 ± 48.5	0.21
Hemoglobin	12 ± 2	12.20 ± 2.07	12.01 ± 1.86	0.65
Creatinine (mg/dl)	1.1 ± 0.7	1.21 ± 0.81	1.03 ± 0.40	0.23
GFR <60 ml/min	32 (28.0)	24 (29.6)	8 (24.2)	0.58
**Echocardiography data**, ***n*****(%)**				
LV EF%	38.3 ± 9.4	39 ± 10	37 ± 9	0.49
Apical ballooning	82 (71.9)	58 (71.60)	24 (72.72)	0.78
Non-apical ballooning	30 (26.3)	22 (27.1)	8 (24.2)	0.79
Mitral regurgation	60 (52.6)	42 (51.85)	18 (54.54)	0.79
Tricspid regurgation	49 (43)	33 (40.74)	16 (48.48)	0.44
RV-Involvement	26 (22.8)	17 (21)	9 (27.3)	0.47
**Medical history**, ***n*****(%)**				
Smoking	36 (31.5)	27 (33.33)	9 (27.27)	0.52
Diabetes mellitus	26 (22.8)	19 (23.45)	7 (21.21)	0.79
BMI>25 kg/m^2^	31 (27.2)	24 (29.62)	7 (21.21)	0.53
Hypertension	66 (57.9)	48 (59.25)	18 (54.54)	0.64
COPD	22 (19.2)	18 (22.22)	4 (12.12)	0.21
Atrial fibrillation	21 (18.4)	16 (19.75)	5 (15.15)	0.56
Coronary artery disease	22 (19.2)	17 (20.98)	5 (15.15)	0.47
History of malignancy	16 (14.0)	10 (12.34)	6 (18.18)	0.41
**Drugs on admission**, ***n*****(%)**				
Beta-blocker	35 (30.7)	22 (27.16)	13 (39.39)	0.14
ACE inhibitor	35 (30.7)	24 (29.62)	11 (33.33)	0.60
Aspirin	29 (25.4)	18 (22.22)	11 (33.33)	0.17
Anticoagulation	7 (6.1)	5 (6.17)	2 (6.06)	0.98

* *p-values for the comparison between group 1 and group 2; SD, Standard deviation; ECG, Electrocardiogram; EF, Ejection fraction; BMI, body-mass-index, COPD, Chronic obstructive pulmonary disease; ACE, Angiotensin-convetring-enzyme*.

### Descriptive Circadian-, Day-, Month-, and Seasonal- Analysis

**Figure 1:**
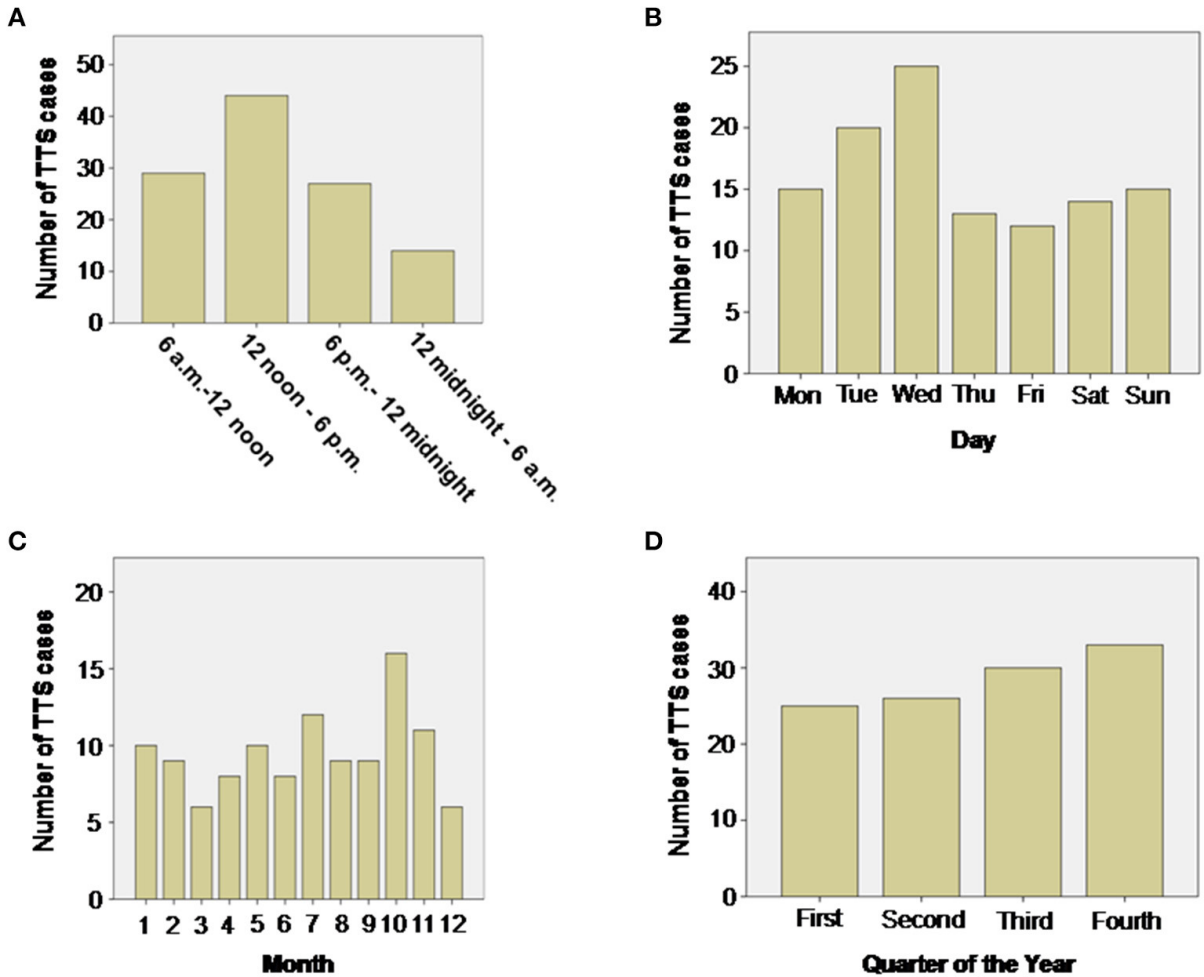
Distribution of TTS events during the day **(A)**, week **(B)**, month **(C)** and quarter of year **(D)**. During the day the number of TTS events show a wave-like pattern with most TTS events taking place in the afternoon between noon and 6 p.m. (*p* = 0.001 Chi-Squared Goodness of Fit Test). No significant differences could be detected by evaluating weeks, months or quarters of year.

The role of diurnal variations influencing the onset of TTS was evaluated by dividing the day into four periods: 6 a.m. to 12 noon, 12 noon to 6 p.m., 6 p.m. to 12 midnight, 12 midnight to 6 a.m. TTS events were most common between 12 noon and 6 p.m. (*n* = 44, 38.5%) and least common between 12 midnight and 6 a.m. (*n* = 14, 12.2%), respectively, depicting a wave-like pattern of occurrence. This distribution was significantly different from the expected equal distribution of *n* = 28.5 TTS cases per 6 h unit (114/4), *p* = 0.001 (Chi-Squared Goodness of Fit Test), Figure 1A.

Although the onset of TTS events over a week-long period was statistically insignificant with an expected equal distribution of *n* = 16.3 TTS cases per day (*p* = 0.25), Figure 1B, most events were recorded on a Wednesday (*n* = 25, 21.9%) and least on Friday (*n* = 12, 10.5%).

A monthly distribution of TTS events revealed that most cases took place in October (*n* = 16, 14%) and the least in March and December (*n* = 6, 5.5%). However, this distribution was not significantly different from the expected equal distribution with *n* = 9.5 TTS cases per month (*p* = 0.67), Figure 1C.

TTS events, grouped quarterly, increased continuously in number with a maximum number of cases diagnosed at the last quarter of the year (*n* = 33, 28.9%), however, this distribution was also not significantly different from the expected equal distribution with *n* = 28.5 TTS cases per quarter of year (*p* = 0.70), Figure 1D.

### Impact of Time Onset of TTS-Event on the Outcome

**Figure 2:**
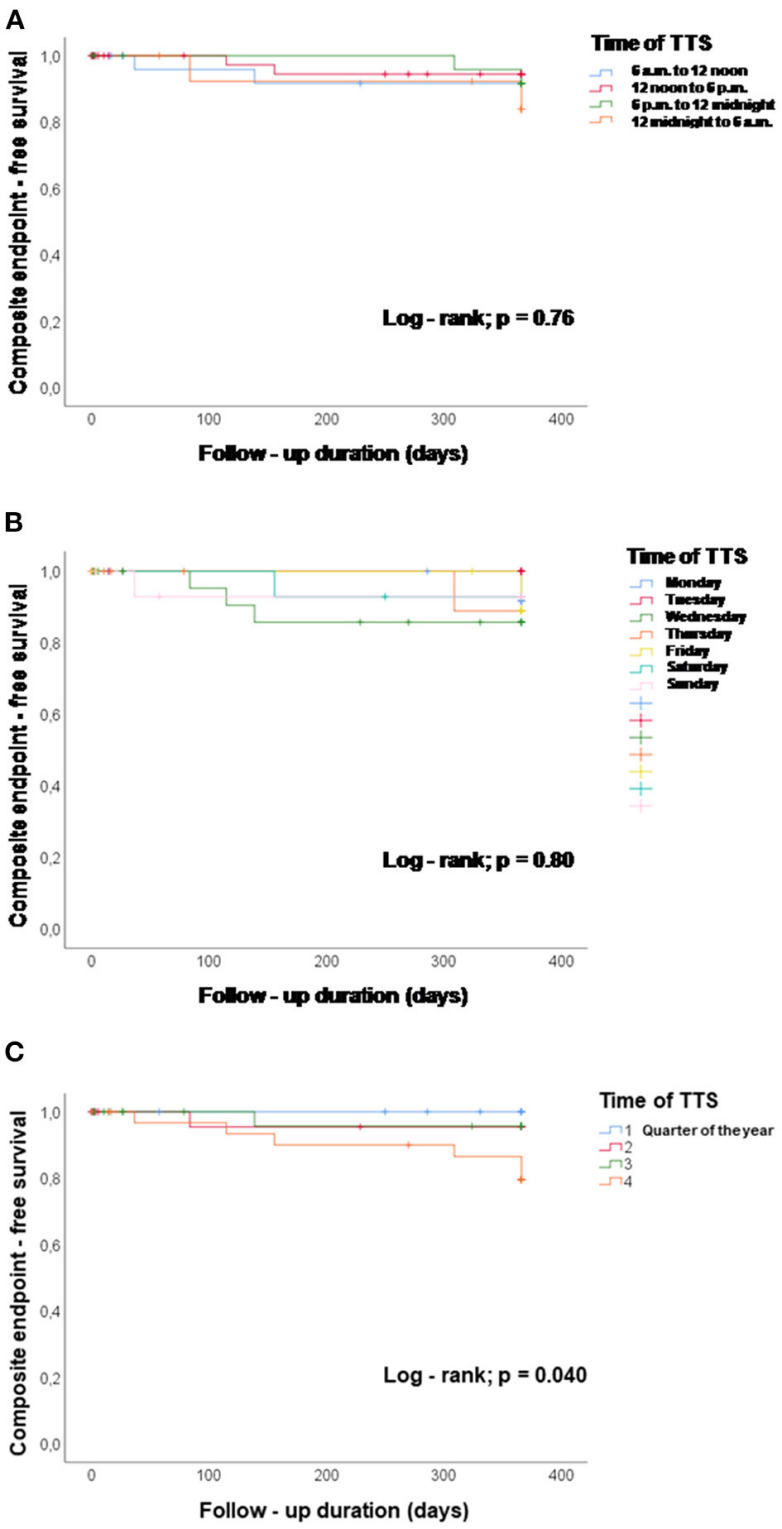
Chronobiological distribution of TTS events and event-free survival (events are defined by the composite endpoint consisting of thromboembolic events, life-threatening arrhythmias, all-cause mortality, re-hospitalization due to heart failure, stroke, myocardial infarction and recurrence of TTS rate). **(A)** TTS events taking place during the four 6 h units of the day were not associated with the event-free survival during 1 year, log-rank *p* = 0.76. **(B)** Time onset of TTS during the day of the week did not impact the event-free survival, log-rank *p* = 0.80. **(C)** Patients with TTS in the fourth quarter of the year showed a lower event-free survival rate compared with the other patients, *p* = 0.04, quarter of year 4 vs. quarters of year 1–3.

**Figure 3:**
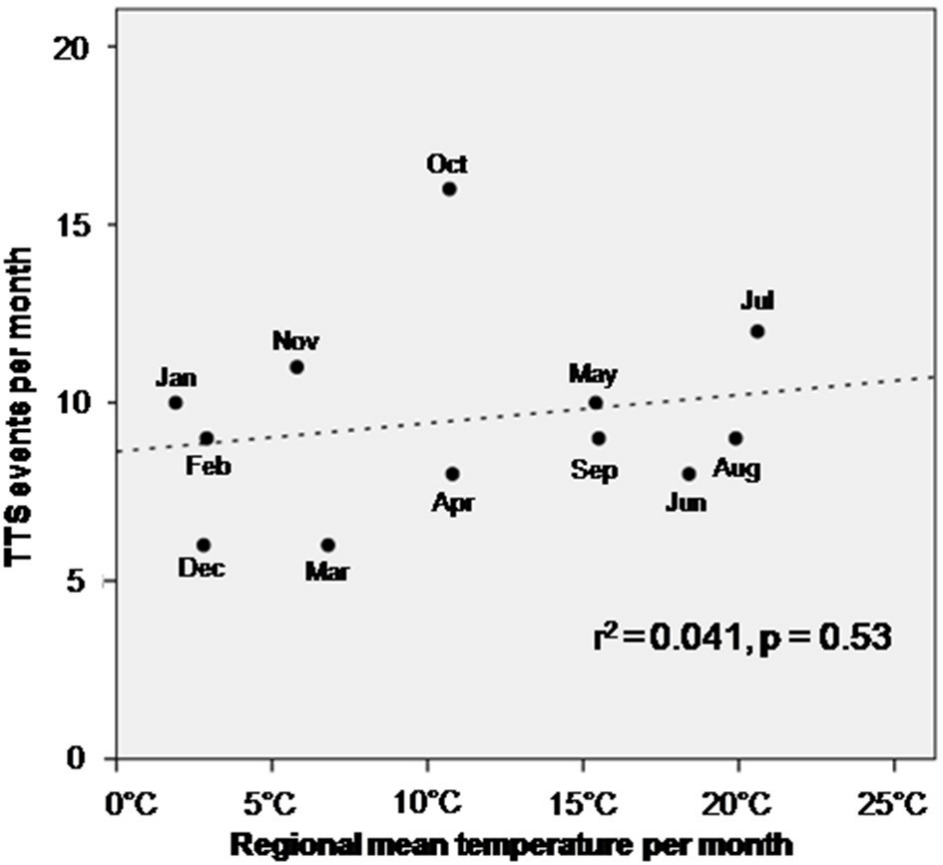
Correlation of temperature per month with the incidence of TTS. No significant association has been observed.

TTS events taking place during the four 6 h units of the day were not significantly different in their association with the composite endpoint after 1 year, log-rank *p* = 0.76, Figure 2A. The composite endpoint included thromboembolic events, life-threatening arrhythmias, all-cause mortality, re-hospitalization due to heart failure, stroke, myocardial infarction and recurrence of TTS. Time onset of TTS during the day of the week also did not impact the outcome (log-rank; *p* = 0.80), Figure 2B. However, patients with TTS diagnosed in the fourth quarter of the year showed a lower event-free survival rate as compared to the other patients (quarter of year 4 vs. quarters of year 1–3; *p* = 0.04), Figure 2C. Regarding seasonal variations of disease incidences, average temperature in each month was presented together with the monthly incidence of TTS. No significant correlation has been found, Figure 3.

### Comparison of November With Other Months

Baseline characteristics of patients diagnosed with TTS in the last quarter were compared to TTS patients from other groups, due to the increased incidence of adverse outcomes in the former.

The values of blood pressure as well as heart rate were not significantly different. ECG-changes like ST-segment elevation and inverted T-waves were present in both groups, without significant difference. However, it was noticed that the QTc interval was significantly longer in TTS patients admitted in the fourth quarter as compared to those patients diagnosed in other months and other quarters (494.59 ± 59.59 ms vs. 472.96 ± 48.26 ms; *p* = 0.05), Table 1. The echocardiographic criteria were also similar in both groups. Additionally, in-hospital complications such as life-threatening arrhythmia, cardiogenic shock, in-hospital death, resuscitation and thromboembolic events were similar in both groups, Table 2.

**Table 2:** In-hospital events in TTS patients with events in the fourth quarter of year.

**Variables**	**All quarters (*n* = 114)**	**Quarter 1–3 (*n* = 81)**	**Quarter 4 (*n* = 33)**	*p*-value^*^
**In-hospital events**				
Life-threatening arrhythmia	13 (11.4)	8 (9.87)	5 (15.15)	0.42
Resuscitation	9 (7.8)	7 (8.64)	2 (6.06)	0.64
Admission to ICU, length of stay	4.4 ± 6.3	4.4 ± 6.7	4.5 ± 5.3	0.86
In-hospital death	9 (7.9)	8 (9.87)	1 (3.03)	0.21
Acquired long QTs	73 (64.0)	48 (59.25)	25 (75.75)	0.02
Cardiogenic shock	22 (19.2)	16 (19.7)	6 (18.1)	0.94
**Treatment strategy**				
NPPV and intubation	39 (34.2)	28 (34.56)	11 (33.33)	0.90
Inotropic agents	21 (18.4)	16 (19.75)	5 (15.15)	0.56
Defibrillator-implantation	1 (0.8)	1 (1.23)	0 (o)	0.52
VA-ECMO	1 (0.8)	1 (1.2)	0 (0)	1.00

* *p-values for the comparison between group 1 and group 2; NPPV, Non-invasive positive pressure ventilation; VA-ECMO, Veno-arterial extracorporal membrane oxygenation; ICU, Intermediate care unit*.

In a Cox univariate analysis of TTS events in the fourth quarter of year (HR 3.04, 95%CI: 1.1–8.3; *p* = 0.03), right ventricular involvement (HR 3.22, 95%CI: 0.8–12.9; *p* = 0.09) and glomerular filtration rate <60 ml/min (HR 3.63, 95%CI: 0.8–16.2; *p* = 0.09) were associated with lower event-free survival rate with the composite endpoint. A multivariate Cox regression analysis was performed with adjustment for the known risk factors, to highlight an independent association with a lower-event free survival rate. This analysis revealed that TTS taking place only in the fourth quarter (HR 6.8, 95%CI: 1.3–35.9; *p* = 0.02) was an independent predictor of lower event-free survival rate, Table 3.

**Table 3:** Univariate and multivariate Cox regression analysis of TTS patients reveal TTS events in the fourth quarter of year as an independent predictor with elevated risk for the composite endpoints during 1 year follow up.

	**Univariate analysis**			**Multivariabe analysis**		
	**HR**	**95%CI**	***P*-value**	**HR**	**95%CI**	***P*-value**
Male	0.84	0.1–6.9	0.87			
Age	1.02	0.9–1.1	0.42			
GFR <60 ml/min	3.63	0.8–16.2	0.09	0.24	0.05–1.2	0.08
Cardiogenic shock	2.24	0.4–11.6	0.33			
Fourth quarter	3.04	1.1–8.3	0.03	6.83	1.3–35.9	0.02
EF ≤ 35%	2.83	0.6–11.8	0.15			
Right ventricular involvement	3.22	0.8–12.9	0.09	1.51	0.3–7.3	0.60
Inotropic drugs	2.03	0.4–10.0	0.38			
DM Typ II	0.42	0.0–3.4	0.42			
Hypertension	1.19	0.3–4.9	0.80			
Apical ballooning	2.83	0.3–23.0	0.33			
Smoking	0.66	0.1–3.3	0.61			

*The composite endpoints consisted of thromboembolic events, life-threatening arrhythmias, all-cause mortality, re-hospitalization due to heart failure, stroke, myocardial infarction and recurrence of TTS rate*.

*HR, hazard ratio; EF, ejection fraction, CRP, C-reactive protein; GFR, glomerular filtration rate*.

## Discussion

Our retrospective clinical investigation of these 114 consecutive TTS patients, has helped us summarize the following; (i) The occurrence of TTS exhibits a circadian rhythm; (ii) the event-free survival rate is significantly lower in TTS patients admitted in the fourth quarter of year; (iii) TTS patients presenting with TTS in fourth quarter of the year show a significant QTc prolongation in comparison to the general TTS population.

Mechanisms dictated by the hypothalamic-pituitary-adrenal (HPA) axis and the sympathetic adreno-medullary system maintain biological homeostasis and protect living organisms from internal and external stresses during environmental and physiological challenges. The HPA axis is activated by corticotropin-releasing hormone (CRH) from the paraventricular nucleus of the hypothalamus, stimulating production of glucocorticosteroids (12). The central biological clock controls the HPA, with cortisol levels maintaining a robust daily rhythm, while circulating levels exhibit an early morning peak.

ACS seems to exhibit some temporal preference in its onset, and is characterized by diurnal variations, variations as per day of the week as well as monthly variations, albeit with analogies and differences. Each of these temporal frames has been discussed in detail (9, 13–19).

A meta-analysis extrapolating data from 30 studies on ACS and 19 studies on sudden cardiac death estimated that nearly 27.7% of morning cases of ACS and 22.5% of sudden cardiac deaths were attributable to an increased risk at that time of day. Recent studies give credence to the hypothesis that there exists a Monday preference for the occurrence of ACS (20). In cases of TTS, however, this data is controversial. Our study suggested a wave-like pattern of occurrence throughout the day with a predominance of TTS events in the afternoon between noon and 6 p.m. and fewer events at night/early morning between midnight and 6 a.m. This data correlates with studies by Sharkey et al. (9, 18, 21) from USA. In that study, the day was divided in 4 h rhythm beginning at 12.00 a.m. In this study the TTS rate was higher presented on Tuesday compared to a higher presence on Wednesday as illustrated in the present study. Additional data from Japan, France, Korea and Italy have presented higher TTS event rates in the morning (8, 9, 18, 21, 22). Kurisu et al. showed a higher presentation of TTS from 06.00 to 06.00 p.m. In that study, the day was divided into six periods in Japan. Song et al. divided the day into four periods and presented a similar result in Korea. Previtali et al. showed also a consistent data of the study group in Japan and Korea. In a study from Italy, the day was also divided into four periods. Data of the present study presenting an afternoon peak of TTS events and its difference to other studies may reflect different habits in different countries. The recruitment of patients in all these studies was in particular similar including patient chart review with retrospective and/or prospective character.

A Monday preference for TTS events has been suggested in studies from Italy and Korea, although our data did show a possible Wednesday preference for occurrence of TTS (increasing from Monday to Wednesday but not reaching significance), more like data reported from studies conducted in the USA (9, 22, 23). Sharkey et al. presented a study suggesting an increased frequency of TTS events occurring in winter. In contrast, studies form Italy and Korea suggest a predominance of TTS events occurring in summer (19, 22). Our data suggested a possible increase in TTS cases reported from the first quarter of year to the fourth with predominance in autumn but without significance. These differences might be linked to differences of the weather in each country.

Increases in plasma catecholamines and cortisol, heart rate, blood pressure, coronary vasomotor tone and platelet aggregation predispose to coronary artery occlusion and this has been correlated to the early morning circadian peak characteristic of ischemic heart disease–related events (24–26). In contrast, the afternoon peak shown here in TTS is consistent with the importance that environmental factors play as triggers in this condition. The predilection of TTS events for early afternoon hours reported here offers some potential insights into the pathophysiology of this condition.

It has been suggested that diurnal variations may have an impact on the clinical outcome of ACS patients, because several fatal cases were diagnosed in the morning, independent of patient age and infarction site or extension (27). A correlate establishing chornobiological variations similarly influencing the outcome of TTS patients can be drawn based on this existing data. TTS patients admitted in the fourth quarter of the year showed a significantly decreased event-free survival rate after a follow-up of 1 year. This finding may support the evidence that the pathophysiology of TTS is still not clearly defined.

Our data offers a detailed analysis of the chronobiological association of TTS events in a German population for the first time, and additionally, detail its influence on the reported adverse outcomes.

This study had a few limitations; firstly, this was a single-center retrospective case series study including patients admitted over the period of 13 years without a comparative control group and therefore there is a need to evaluate results in large multicenter registries. Secondly, data was analyzed in an exploratory manner to generate new hypotheses concerning time-dependency of TTS events and associated adverse events. Therefore, we cannot rule-out random or accidental findings. The timing of onset of TTS may vary because some patients who had a symptom at night might come to the hospital on the next day.

## Clinical Perspectives and Translational Outlook

TTS seems to exhibit temporal preference in its onset. The chrono-biological distribution of TTS may influence the outcome of patients. These novel findings may help to identify high risk TTS patients with a need of subsequent follow-ups.

## Data Availability Statement

The data analyzed in this study is subject to the following licenses/restrictions: original data are available under request according to the ethical committee. Requests to access these datasets should be directed to ibrahim.el-battrawy@medma.uni-heidelberg.d.

## Ethics Statement

The studies involving human participants were reviewed and approved by MA 2018/KA. The patients/participants provided their written informed consent to participate in this study.

## Author Contributions

IE-B, AA, SL, XZ, UA, MB, and IA designed the research and wrote the paper. IE-B, IA, and TG performed the clinical research. IE-B, SL, AM, XZ, NU, TG, and IA analyzed data. All authors contributed to the article and approved the submitted version.

## Conflict of Interest

The authors declare that the research was conducted in the absence of any commercial or financial relationships that could be construed as a potential conflict of interest.

## Publisher's Note

All claims expressed in this article are solely those of the authors and do not necessarily represent those of their affiliated organizations, or those of the publisher, the editors and the reviewers. Any product that may be evaluated in this article, or claim that may be made by its manufacturer, is not guaranteed or endorsed by the publisher.
